# An Evening with Bonwill

**Published:** 1900-04

**Authors:** I. Norman Broomell


AMERICAN JOURNAL

—OF—

DENTAL SCIENCE.

VOL. XXXIII. BALTIMORE, APRIL, 1900. NO. 12.

### Selections.

ARTICLE I.

### AN EVENING WITH BONWILL.

I. NORMAN BROOMELL, D. D. S.

"Good evening, young man. I have come to show you how to 'grind on' a set of teeth according to geometrical and anatomical laws."

Such were the words spoken by the subject of this sketch, as, passing his fingers through his gray locks, he placed his familiar grip at my feet. The time was an evening late in July of the past year, and the occasion one long anticipated and long to be remembered by me.

"Now, let us take off our coats and get to work, as I must be getting back home; I have many things to do before 2 a. m. Did I ever tell you that I do the biggest part of my brain work after the rest of you young fellows are in bed?"

"Are you using the articulator I gave you?" A nod in the affirmative was met by, "I don't believe you, or else you would have sent for the extra bows; I always keep them back to catch skeptical boys like you."

"What do you use such stuff as that for? Not for a base plate! Throw it away," putting his own command into violent execution; this supposed ideal product of the dental material makers was soon replaced, with the remark, "I prepare this kind myself."

"Now give me a fair-sized wheel. This is carborundum; why don't you use corundum? Here is an upper and a lower set of teeth; I will grind them on and I will leave you this other set to do yourself, and I will bet you can't do it. There is not a man in this city that can do it. Not even the professors who try to teach prosthetic dentistry.'

"Why do you always—?" "Hold on now, wait until I am through; I want to do the talking; you listen. Now you see I take these upper incisors and grind off the inner side of the cutting edge, making a bevel there of about forty-five degrees, and place them back again on the card. Now this is the first thing I always do in articulating a set of teeth. What are you laughing at? 'Articulation' is right, it is not 'occlusion.' Occlusion will do when referring to closing the lips or mouth, but it does not apply to the movements of the lower jaw through the action of the tempero-maxillary joint. If the action of the lower jaw were directly up and down, we might refer to the closing of the teeth as occlusion." "Wait a minute—" "Now what is it? Remember, I said no interruptions; but go ahead, speak up!" "You acknowledge that the movement of the lower jaw is not the same in all cases, and that it is seldom directly up and down." "Yes; well, what of it?" "I was wondering why it is, if the movements of the jaws are not always the same, that you grind the occlusal surfaces of the teeth alike in all instances." Almost before I had finished came the reply: "To compel them to be all alike, as the Almighty intended they should be. When you ask that question, it shows how little you know of the fundamental principles governing the teeth in man."

"Give me a pair of dividers. What! you haven't any? What would you think of a carpenter who would try to work without a saw? Well, dividers are just as essential in a dentis's hands. Did I ever show you how to square a circle? Ha, ha, ha! Well, I can come very near doing it, but never mind about that now. Don't sit down, stand up here; I want you to see how I grind these bicuspids. I have stood up to 'grind on' teeth for over forty years and I feel younger to-day possibly than you do."

After thus relieving himself of an opinion doubtless based upon his own physical convictions, he took from the card, one at a time, the bicuspid teeth belonging to the upper set, and with all the enthusiasm of the most ardent student, cut upon the occlusal surface of each, from mesial to distal, a well-defined, rounded groove, converting this surface into a double inclined plane extending from buccal to palatal.

"Now, do you see the way the bicuspids come together? They meet with all their surfaces touching." Then, while rotating the articulator from side to side, and looking in from the back, as in viewing the mouth from the pharynx, he added with enthusiasm: "Now, isn't it beautiful! And so perfect mathematically; after a preordained and established law; nothing could give a better idea of the wisdom of the Almighty. Now, the fact that I grind these teeth this way—you see I am doing the same thing with the molars—is not my creation, I am simply carrying out Nature's law as found in the human jaws, and having as its basis an equilateral triangle."

By the time my visitor had completed the grinding of the upper teeth, all of which, so far as the posterior teeth were concerned, were treated in a similar manner, he appeared completely wrapped up in what he was doing, and the energy of his toil was only surpassed by his desire to have me fully comprehend what he was doing and saying. At one time he looked up from his work long enough to ask if he was using too many "I's." After a glance, he appeared to realize that his remark was understood, and, referring to the incident which called it forth, he said: "That Western editor who had sufficient leisure to count the number of I's used by me in the publication of my personal experiences during my trip to Europe in 1889 taught me a lesson, but it was not one by which I could profit; the work which I have been doing in dentistry for nearly half a century has been in a special line, and of rather a unique character; I have not imitated or even referred to the works of others. All that I have done for dentistry has been after my own ideas of what was right; *theories* at first, facts soon afterward. Advice, although frequently proffered, was seldom accepted, this (pointing to his forehead) being my confidential and most reliable adviser. I was compelled to use the personal pronoun then, and shall continue to do so; when all your thoughts and actions are your own, it is impossible to do otherwise." After nervously consulting his time piece, he continued his work by taking the lower incisor, grinding from the labial cutting edge the round smooth surface common to finished porcelain. This grinding was so slight and performed so quickly, that it appeared to have no practical bearing on his theory.

"Now, the proper width for these lower incisors I get with the dividers after the plans laid down in this little chart" (here unfolding a sheet filled with many complex drawings); "by placing the dividers at this point I draw a line from A to B, and from C to D we get the radius of another arc intersecting with the line previously drawn from A to B; all within this equilateral triangle; now is that clear to you?" With a desire to avoid unhappiness, I had always made it a point to agree with Bonwill, so I answered in the affirmative. "Now with the proper manipulation of the dividers we get many equilateral triangles within the main triangle, each one of which denotes the exact size and location of one of the teeth in each jaw."

Venturing another question, I said: "You always take four inches as the size of the primary equilateral triangle?" "Yes." "And always go through the same geometrical figuring to obtain the position and width of each individual tooth?" "Yes." "And always grind the teeth in about the same manner before mounting them in the base plate?" "Yes; what of it?" "Do you not find that you get practically the same results in all cases? Do you fail to recognize a marked variation in the movements of the mandible, in some directly up and down, in others a more or less lateral motion?" After a hearty laugh, he said: "Of course I know it, to be sure I do; there are almost as many movements as there are jaws, but there is only one normal movement of the lower jaw, only one intended by the Creator—that is, the movements of all human jaws should be the same; why, how could we consider it any other way, when we see so beautiful and so mathematical a design employed by the Creator in the construction of the lower jaw? There is only one way by which the exact law governing the movement of the lower jaw can be carried out, and that is to articulate the teeth in such a manner that they will compel a perfect mechanical movement of the mandible, and in this way and in no other you will have perfect mastication."

"Now you see in grinding the lower bicuspids and molars I have intentionally refrained from cutting off from the lingual side. I do this because the teeth are always made with their lingual cusps too short; they should be made longer, and the only way to overcome this is by grinding from the buccal cusps alone."

Bonwill now proceeded to arrange the teeth more carefully in position on the base plate, placing one at a time and viewing it in every direction, his face taking on an expression of profound admiration as he said: "Now look at that; could you imagine anything nearer perfection? All that Nature wants is a chance to right herself, and with this beautiful arrangement of the articulation of the teeth we do a great deal to assist her in this direction.

This reminds me of the boy in my laboratory; did I ever tell you about him? It took him a long time to get on to the method of mounting teeth according to my rules, which are Nature's rules, notwithstanding he was apt and quick. One day I took a case from his hands and said, 'let me do that; you're getting it wrong.' When I returned them to him, he was quick to observe an intended imperfection which I had left. Taking them again, I corrected the defect and handed him the case. Instantly he said, 'that's it, that's it; why Doctor, if there is any such a thing as a God, He couldn't do better than that." While I was unable to see the force of this incident, Bonwill evidently thought it carried great weight, referring to it on numerous private occasions as well as in his occasional lectures to students.

By this time (11.30) I was so engrossed with Bonwill's whole souled desire to have me understand as he understood, to think as he thought, that I determined to get more out of him, not particularly in the line of the articulation of teeth, but in matters of general practice. In the meantime a little lunch had been provided in the dining room, and of this my visitor was invited to partake. His ready acceptance without even referring to the time of night encouraged me in the belief that he had more to say. After being seated, about the first thing he said was, "well, what do the boys think of me by this time? I wish I knew; well, I think I do know; but we will not talk about that now. Before it gets too late I want to show you how I pack amalgam." He had evidently anticipated this demonstration, for as he spoke the words he reached down and took from his grip all the requisites necessary to put his remark into execution. "Here is a lot of amalgam for you; I don't sell this, I give it away for what it costs to make it. I do this because I want everybody to use it, and I want everybody to use it because I know it will save teeth better than anything else. You fellows don't know how to use amalgam, that's the reason it fails, and then you think you can get more money for gold; ah! that's what most of you are after, the money, the money; it isn't the desire to save teeth. Now, with this I can do both; I can save teeth and I can get as much money for it as by using gold. Why do we impose a fee? Not for the material used, but for the service rendered, and that service means the saving of teeth."

Simultaneously with this talking Bonwill was "packing amalgam," as he termed it, using for a cavity a steel plate in which were drilled a number of holes of various sizes, these passing entirely through the plate. In mixing the alloy he used the palm of his hand for a mortar, and with the ball of his thumb for a pestle the mass was amalgamated with true Bonwill enthusiasm. The amalgam was then squeezed only moderately dry and dropped into the metal cavity in quite large pieces. Then came the most interesting part of the demonstration; grasping a plugger (one of his own design, of course, because Bonwill seldom made use of those planned or designed by others), he proceeded to force the alloy into the cavity. Force is the only expression to use in describing his methods.

The heavy handle of the plugger was taken by the hand of the operator—not by the finger and thumb, but by the entire hand; in this way bringing into action the wrist muscles, the plug receiving the combined force from the hand and wrist at each punch or push of the instrument. It might be added that the force above referred to was just as much a factor during operations in the mouth, and anyone who has had the privilege of seeing some of Bonwill's amalgam work can testify to the value of the methods which he employed.

"See, the cavity is now almost full, and I am bringing all the surplus mercury to the surface by pressure on this bibulous paper, which absorbs it. In the bottom of the filling there is no mercury and the metal is almost as hard at that point as it will ever be."

After turning over the metal plate, I found this to be the true condition; the filling was already so hard that no impression could be made upon it with the sharp point of an instrument.

"Now as I approach the surface I take up the surplus mercury by the addition of fresh alloy, rubbing it in with this round burnisher; rub it hard, then add more filling and rub again; keep this up until the surface of the filling is as hard as the bottom."

"Now it is finished, and I will show you what a perfect mass I have made," and taking an iron punch and a hammer, he proceeded to force the plug from the hole in the steel plate, which was accomplished only by a series of heavy blows. "There, it is almost as hard and compact as the steel plate itself." And so I found it; the entire operation, which had occupied but a few minutes, had resulted in the production of a metal plug which, in the mouth, would defy the force and strain of mastication. "Why, it's nearly one o'clock! I must get out. Wish I had time to tell you more about filling teeth with amalgam and the way I form a matrix for the purpose, by using a rubber-dam clamp and modeling composition or gutta-percha; also how I use Abbey's old-fashioned gold foil, and the use of paraffin in connection with oxyphosphate. But you're like everybody else, you don't want to listen. Good-night. I'll see you at Asbury Park."—*Dental Brief.*

